# Development and Evaluation of a Water Soluble Fluorometholone Eye Drop Formulation Employing Polymeric Micelle

**DOI:** 10.3390/pharmaceutics10040208

**Published:** 2018-10-28

**Authors:** Gyubin Noh, Taekwang Keum, Jo-Eun Seo, Jaewoong Choi, Bastola Rakesh, Lamichhane Shrawani, Byoungduck Park, Young Wook Choi, Sangkil Lee

**Affiliations:** 1 College of Pharmacy, Keimyung University, 1095 Dalgubeol-daero, Dalseo-gu, Daegu 42601, Korea; rhgyubin@naver.com (G.N.); gtk02@hanmail.net (T.K.); joeun0405@hanmail.net (J.-E.S.); 79182@naver.com (J.C.); bastola777rakesh@gmail.com (B.R.); phr.shrawani@gmail.com (L.S.); bdpark@kmu.ac.kr (B.P.); 2 College of Pharmacy, Chung-Ang University, 221 Heuksuk-dong, Dongjak-gu, Seoul 06974, Korea; ywchoi@cau.ac.kr

**Keywords:** Soluplus^®^, fluorometholone, polymeric micelle, solid dispersion, nanomedicine, ocular drug delivery

## Abstract

Low aqueous solubility of drug causes difficulties in preparation and inconvenience of administration. Polymeric micelles of fluorometholone (FML) using solid dispersion technique were prepared to develop an eye drop formulation with enhanced water solubility. Solid dispersions of FML were prepared at various FML:Soluplus^®^ *w*/*w* ratios using solvent evaporation method. A physical mixture was also prepared. Physicochemical characterization was performed with various methods. Ex vivo porcine corneal permeation of polymeric micelle, physical mixture, and commercial product were compared. FML solid dispersion (1:15) showed the highest solubility, which was c.a. 169.6- and 15.3-fold higher than that of pure FML and physical mixture. Characterization showed that the crystalline form of FML changed to amorphous state and polymeric micelles were formed in round micelle. Flucon^®^, a commercial product of FML, showed significantly large particle size and high poly dispersity index. In contrast, FML polymeric micelle showed submicron size with uniform size distribution. Ex vivo porcine corneal permeation study showed that permeation by polymeric micelles was significantly higher than that by the commercial product and physical mixture. In addition, confocal laser scanning microscopic analysis supported the enhanced porcine corneal tissue permeation property of polymeric micelle. In conclusion, polymeric micelle prepared with solid dispersion using Soluplus^®^ can be a potential nanomedicine for ocular delivery of poorly water-soluble FML.

## 1. Introduction

The most common technique of applying drugs to the eyes is to directly apply the ocular formulation on the surface of the eye. Eye drops are the common form of formulation for anterior segment disorders; however, they are easily washed away by tears within 0.5–1 min after application [1]. Therefore, the duration of the effect of a drug on the ocular surface is very short [2]. In addition, the eyes have protective functions and structural features to maintain their normal condition because they are exposed to the external environment. This makes it difficult to deliver the drug to the desired site resulting in low drug bioavailability [3]. Thus, only less than 5% of the administered dose of eye drops affects the eyes [4]. 

Fluorometholone (FML) is a corticosteroid drug that is commonly used for inflammatory diseases and dry eye syndrome [5,6]. The preparation of FML solution is difficult owing to its low aqueous solubility [7]. The commercially available FML formulation is a suspension (e.g., Flucon^®^, Alcon Laboratories Pty. Ltd., Fort Worth, TX, USA). FML in suspension shows low bioavailability of around 1% and causes difficulty in delivering the accurate dose, leading to inconvenience in administering the suspension to the eye [8]. Therefore, excessive doses are often administered to achieve the desired effect and such dosing regimens may cause side effects, such as elevated intraocular pressure [9].

Various ocular drug delivery systems have been studied to improve low bioavailability caused by the unique structure of eye and to increase the solubility of poorly water-soluble drugs (Table 1) [10,11,12]. To date, various formulations, such as emulsions, ophthalmic ointments, suspensions, hydrogels, nanoparticles, liposomes, dendrimers, ocular inserts, and contact lens formulations, have been studied to overcome drug permeation barrier of the ocular drug delivery system [11,13,14,15]. Among them, micelles have a relatively simple preparation process and can form carriers that have high drug loading efficacy as well as small and homogeneous particle size. Therefore, micelles can be used to dissolve poorly water-soluble drugs to make transparent aqueous formulations [14].

Cyclodextrin derivatives such as hydroxypropyl β-cyclodextrin and sulphobutylether β-cyclodextrin complex were used to solubilize FML [16,17] and FML ocular inserts using poly(dl-Lactic acid) (PLA) and poly(dl-lactic-co-glycolic acid) (PLGA) polymer were studied [18]. Nevertheless, no method has been reported to sufficiently increase the water solubility of FML with good physical stability. 

**Figure 1:**
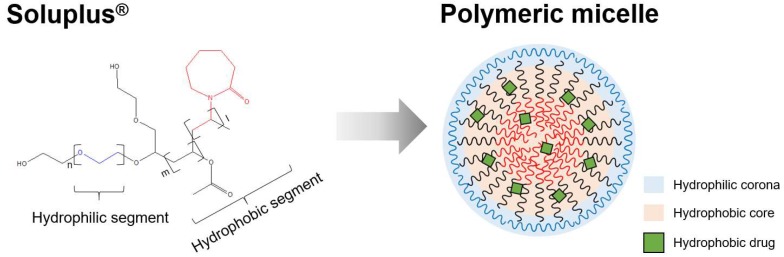
Concept of polymeric micelle prepared with Soluplus^®^. The red, black and blue segments are polyvinyl caprolactam, polyvinyl acetate, and polyethylene glycol, respectively.

Soluplus^®^ is triblock-copolymer of polyvinyl caprolactam, polyvinyl acetate, and polyethylene glycol. It has average molecular weight of 118,000 g/mol [19]. It is an amphiphilic copolymer comprising hydrophilic and hydrophobic blocks (Figure 1). Owing to its amphipathic nature, it is self-assembled in aqueous media to form a polymeric micelle with core-shell structure. Also this polymer has the advantage that it forms micelles in low concentration due to its low CMC (7.6 mg/L) [19]. The hydrophilic corona of polymeric micelle stabilizes and solubilizes the micelles in aqueous environment, and the hydrophobic core incorporates and protects hydrophobic drugs [20]. In addition, this polymer is known to serve as the matrix of solid dispersions to assist dissolution of drugs [21,22]. Through this mechanism, Soluplus^®^ can enhance the water solubility of various biopharmaceutical classification system (BCS) class II and IV group drugs [23]. Soluplus^®^ was added to the oral drug delivery system of poorly water-soluble drug dutasteride to increase physicochemical stability and bioavailability [24]. Itraconzole/Soluplus^®^ tablet showed improved solubility, AUC, and *C*_max_ [25]. Moreover, Soluplus^®^ improved AUC and *C*_max_ of BCS class II drugs such as danazol and fenofibrate [21].

To date, few attempts have been made to increase the water solubility of poorly water-soluble drugs using Soluplus^®^. The polymeric micelles encapsulating α-lipoic acid showed improved corneal permeability through increased solubility of drug [26]. Also curcumin encapsulating polymeric micelles improved in vivo corneal permeation and showed higher anti-inflammatory activities than free curcumin [27]. In this study, polymeric micelle of Soluplus^®^ incorporating FML was fabricated, and the physicochemical characteristics and permeation enhancing effect were evaluated to prove the feasibility of FML/Soluplus^®^ as an eye drop formulation.

## 2. Materials and Methods 

### 2.1. Materials

FML was purchased from Wako pure chemical industries Ltd. (Osaka, Japan). Soluplus^®^ was purchased from BASF SE (Ludwigshafen, Germany). Coumarin-6 was purchased from Sigma-Aldrich (Saint Louis, MO, USA). All other chemicals and solvents were of reagent grade.

### 2.2. Methods

#### 2.2.1. Preparation of Solid Dispersions and Physical Mixture

The solid dispersions were prepared by solvent evaporation method at various FML:Soluplus^®^ *w*/*w* ratios (1:7, 1:10, 1:15). FML and Soluplus^®^ were added to a round flask, followed by the addition of a minimum volume of ethanol. Then, the mixture was shaken in a water bath at 30 °C for 30 min to solubilize FML and Soluplus^®^. Ethanol was removed by evaporation using rotary evaporator to make a solid dispersion film. Finally, the film was dispersed in phosphate-buffered saline (PBS) to prepare polymeric micelles in aqueous solution. In addition, a physical mixture was prepared with mortar and pestle at a 1:10 ratio.

#### 2.2.2. Analytical Method

FML was analyzed by HPLC with a UV detector. HPLC analysis was performed using Agilent^®^ 1260 infinity LC system. The reverse phase column was Gemini^®^ 5 μm NX-C_18_ 110 Å, LC Column 250 × 4.6 mm (Phenomenex, Torrance, CA, USA). The mobile phase comprised 70% methanol and 30% distilled water. The flow rate was 0.5 mL/min, and the detection wavelength was set at 280 nm. The temperature of column was maintained at 25 °C.

#### 2.2.3. Characterization of Solid Dispersion

##### Particle Size Measurement

An aqueous solution of Soluplus^®^, three different ratios of solid dispersion formulations (1:7, 1:10, 1:15), physical mixture, and the commercial product of FML suspension (Flucon^®^ eye drop 0.1%, Alcon Laboratories, Fort Worth, TX, USA) was prepared for particle size measurement. The particle size was measured using a dynamic light scattering spectrophotometer (ZetaPALS, Brookhaven instruments, Long Island, NY, USA). Samples were diluted in PBS with care to avoid dilution below the critical micelle concentration (CMC) of Soluplus^®^.

##### Encapsulation Efficiency (EE)

The encapsulation efficiency was evaluated according to centrifugation ultrafiltration method [28]. Briefly, a total 0.5 mL of each formulation was added to centrifugal filter device (Amicon^®^ Ultra centrifugal filters 0.5 mL–3 K, Millipore, Burlington, MA, USA). Free FML was separated with centrifugal force of 8000× *g* for 10 min. Then the amount of free FML was quantified by established HPLC method. The encapsulation efficiency was calculated using the following equation, where *W_free_* is the amount of free FML and *W_total_* is the added amount of FML.

$$\;\mathrm{EE}\left(\%\right)=\left(1-\frac{W_{free}}{W_{total}}\right)\times 100\%\;$$

##### Differential Scanning Calorimetry (DSC)

Thermal analysis of FML, Soluplus^®^, solid dispersions, and physical mixture was performed using DSC 4000 differential scanning calorimeter (Perkin-Elmer, Waltham, MA, USA). Ten milligrams of samples were used for DSC. Temperature scans were performed in the temperature range 25–350 °C at a heating rate of 10 °C/min, under a nitrogen purge gas flow of 20 mL/min.

##### Powder X-ray Diffraction

Powder X-ray diffraction (PXRD) analysis was performed using Rigaku D/Max-2500 (Rigaku, Tokyo, Japan). The scanning angle ranged from 3 to 45° at the rate of 0.05°/s. 

##### Fourier Transform Infrared Spectroscopy

Fourier transform infrared (FT-IR) spectra were obtained using Nicolet iS10 FT-IR Spectrometer (Thermo Fischer Scientific, Waltham, MA, USA). The spectra of samples were scanned over a frequency range of 4000 cm^−1^ to 400 cm^−1^, with a resolution of 4 cm^−1^.

##### Transmission Electron Microscopy Imaging

Transmission electron microscopy (TEM) imaging was performed to characterize polymeric micelles using H-7600 transmission electron microscope (Hitachi, Tokyo, Japan), with an accelerating voltage of 100 kV. The samples were stained with 2% phosphotungstic acid, placed on a copper grid, and exposed under infrared lamp for 10 min.

##### Measurement of Solubility of Fluorometholone

The solubility of FML was measured according to Higuchi and Connors’ method [29]. The pure drug and three different ratios of solid dispersion (1:7, 1:10, 1:15) with 200 mg of FML and different amounts of Soluplus^®^ were added to round flasks with 10 mL PBS. Then, sealed round flasks and commercial product were placed in a water bath and shaken at 37 °C for 48 h. Each formulations were filtered through a 0.45-μm nylon membrane filter, and FML dissolved in the filtered solutions was quantified by HPLC.

#### 2.2.4. Ex Vivo Permeation Study

Porcine eyes were obtained from a local slaughterhouse, and the eyeballs were immediately collected after the pigs were sacrificed. The eyeballs were transported in cold PBS solution. Only the eyeballs that were transparent and had undamaged cornea were selected. Then, the corneal tissues were removed with scalpels and scissors, washed with PBS, and stored in normal saline at 4 °C until permeation study. 

Ex vivo permeation study using porcine corneal tissue was performed with Franz-diffusion cell having an effective diffusion area of 0.672 cm^2^. The receptor chamber was filled with PBS (pH 7.4), and the receptor medium was stirred at 600 rpm. Porcine corneal tissues were carefully placed on a Franz-diffusion cell, and 300 μL of each formulation (commercial product, physical mixture, and solid dispersion) was applied to the donor chamber. All formulations contained 0.1 *w*/*v* % of FML. Franz diffusion cell was maintained at 37 °C with a thermostat. A 0.5-mL sample was taken every hour from the receptor chamber for 5 h and immediately replenished with an equal volume of PBS. The permeability of the drug in each formulation was evaluated by plotting the cumulative permeated amount of FML per unit area (μg/cm^2^) over time (h). The steady-state flux (*J_s_*) across the porcine corneal tissue was calculated from the slope of linear portion of the cumulative permeation graph using the following equation:

(1) $$\;J_{s}=\frac{Q_{t}}{A\cdot t}\left(\mu g\cdot {\mathrm{cm}}^{-2}\cdot h^{-1}\right)\;$$

where *Q_t_* = quantity of fluorometholone crossing membrane (in μg), *A* = effective diameter (cm^2^) and *t* = time of exposure (in h). After 5 h, the part of the corneal tissue in contact with the formulation was separated. The separated corneal tissues were minced, placed in a conical tube with 1 mL of methanol, and stirred overnight to extract the deposited FML. The samples were centrifuged the next day, and the supernatant containing FML was collected. All samples were diluted appropriately with methanol and analyzed by HPLC. Each experiment was repeated in triplicate.

##### Imaging Study Using Coumarin-6 as a Fluorescent Probe

Ex vivo permeation study was performed using coumarin-6 (C_6_) as a fluorescent probe to evaluate the permeation enhancing effect of polymeric micelle. C_6_ is a fluorescent probe and suitable as a model of poorly water-soluble drug, because it precipitates in water and forms a microcrystal resulting in significant loss of fluorescence intensity; however, it emits fluorescence when solubilized [30]. C_6_ was dispersed in PBS at a concentration of 50 μg/mL to prepare an aqueous solution. Polymeric micelles containing C_6_ were prepared by same method as described above, where the concentration of C_6_ was same as the concentration of FML in the 1:15 formulation.

The permeation study was performed using Franz diffusion cell. Various formulations were loaded to the donor chamber and the corneal tissues were separated 0.5, 1, 2, and 4 h after loading. The porcine corneal tissue was fixed with OCT compound (Sakura Finetek, Tokyo, Japan) and frozen in liquid nitrogen. The block was cut into 10-μm thick sections using a cryostat (Cryotome FE, Thermo Fisher Scientific, Waltham, MA, USA) and mounted onto slides and cover slipped.

Confocal laser scanning microscopy (Zeiss, Oberkochen, Germany) was used to image the permeation of C_6_ through the porcine corneal tissue. C_6_ in porcine corneal tissues was observed at excitation and emission wavelengths of 488 and 505 nm, respectively.

## 3. Results

### 3.1. Characterization of Solid Dispersion

#### 3.1.1. Particle Size Measurement and Encapsulation Efficiency

The particle size of commercial product was much larger than that of other formulations and showed relatively large deviation (Table 2). In contrast, Soluplus^®^ and solid dispersions were homogeneously dispersed and had smaller particle size than the commercial product (CP) and physical mixture (PM). The particle size increased after encapsulation of FML. As the proportion of Soluplus^®^ increased, the particle size decreased slightly. The particle size of physical mixture ranged between that of solid dispersion and commercial product, and this may be because of the limited solubilization effect of physical mixture. Poly dispersity index (PDI) can be a criterion for even particle size distribution. PDI less than 0.1 indicates good uniformity, while values greater than 0.3 indicate irregular particle size distribution. Based on our PDI measurements, we assumed that the polymeric micelle formulations prepared by solid dispersion method had an appropriate size distribution with physical stability. Nearly all FML was encapsulated into polymeric micelles in all SD groups. On the other hand only a small amount of FML was encapsulated in the PM groups.

#### 3.1.2. Differential Scanning Calorimetry (DSC)

**Figure 2:**
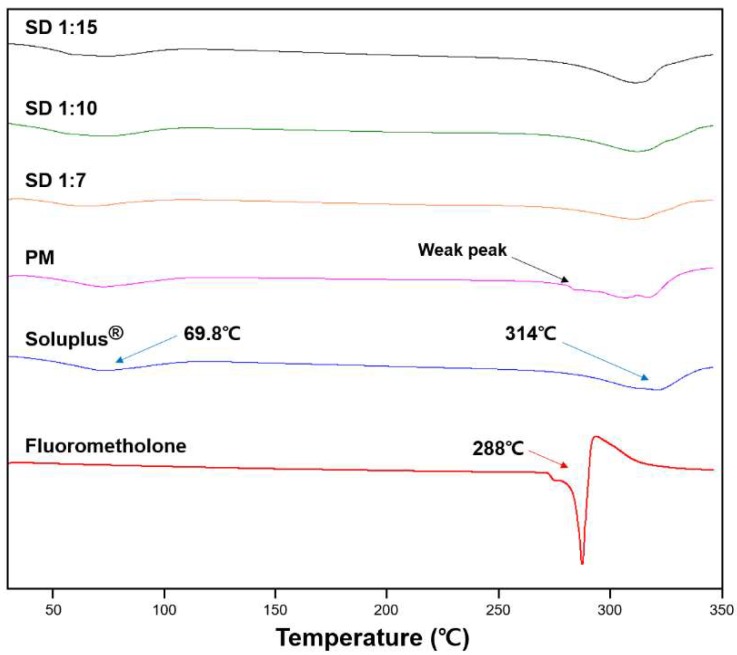
Differential scanning calorimetry thermograms of fluorometholone, Soluplus^®^, and various formulations.

DSC analysis was performed to observe the phase transformation of FML during solid dispersion preparation. As shown in Figure 2, free FML showed a sharp endothermic peak at 288 °C corresponding to the melting point of FML. Soluplus^®^ showed a weak broad endothermic peak at 69.8 °C, corresponding to the glass transition of Soluplus^®^ [19]_._ The other peak at 314 °C may be attributed to the decomposition of the polymer. The thermal profile of solid dispersions showed no characteristic endothermic peak of FML. This might be due to the phase transformation of FML from a crystalline form to an amorphous form or the dissolution of FML into Soluplus^®^ [31]. However, the thermal profile of the physical mixture showed that the weak endothermic peak may correspond to undissolved crystalline FML in the matrix [32]. Therefore, it can be suggested that FML was completely converted from crystalline form to amorphous form in solid dispersion, unlike the physical mixture.

#### 3.1.3. Powder X-ray Diffraction

**Figure 3:**
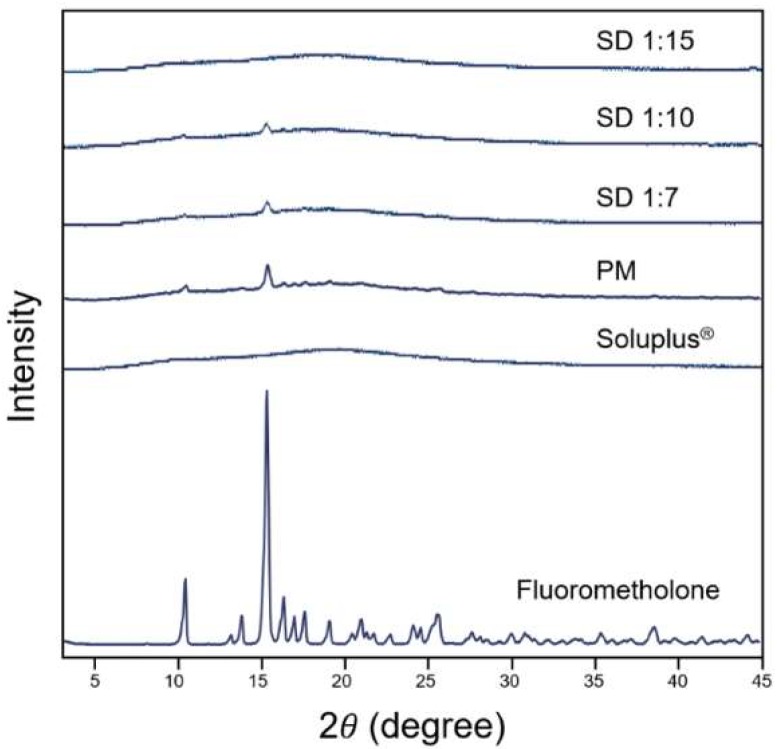
X-ray diffractograms of fluorometholone, Soluplus^®^, and solid dispersion formulations.

To evaluate the physical characteristics of the solid dispersion formulation further, PXRD analysis was performed on FML, Soluplus^®^, and solid dispersions. As shown in Figure 3, FML showed characteristic peaks around 15°, indicating that it was in crystalline form. Meanwhile, the diffractograms of solid dispersions were close to that of Soluplus^®^. A weak peak was found at approximately 15° for the physical mixture and solid dispersions at 1:7 and 1:10 ratios, but a sharper peak was observed for the physical mixture than for the solid dispersions. However, no peak was observed for the 1:15 formulation. These findings indicate that free FML was changed from crystalline form to amorphous form in solid dispersion, as indicated in the thermograms.

As shown in the DSC thermograms, FML has a high melting point of around 288 °C, indicating its strong crystal lattice energy. Because of this feature, FML dissolution requires large energy and has poor water solubility [33]. The above results show that Soluplus^®^ acts as a solid matrix to form a solid dispersion with FML during its transformation from crystalline form to amorphous form. Owing to the low lattice energy of amorphous form of FML in solid dispersion, the solubility can be increased [32].

#### 3.1.4. Fourier Transform Infrared Spectroscopy

**Figure 4:**
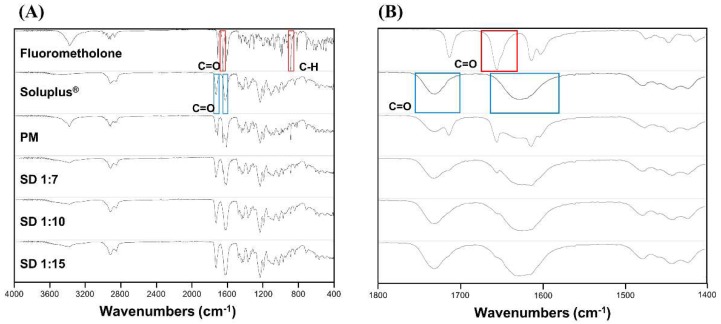
FT-IR spectra of (**A**) range from 4000 cm^−1^ to 400 cm^−1^ and (**B**) range from 1800 cm^−1^ to 1400 cm^−1^ of fluorometholone, Soluplus^®^, and fluorometholone-loaded solid dispersion formulations.

FT-IR analysis was performed to investigate the possible interactions between FML and Soluplus^®^ in solid dispersion. As shown in Figure 4, free FML showed characteristic infrared absorption bands at 887.3 and 1657.4 cm^−1^ corresponding to C–H bending and C=O stretching, respectively. Soluplus^®^ showed peaks at 1628.54 cm^−1^ and 1730.98 cm^−1^ corresponding to two different C=O stretching. The IR spectrum of the physical mixture showed characteristic sharp peaks of FML, indicating that the crystalline form was still present. However, the characteristic peaks of FML and new peaks were not shown in the spectrum of solid dispersions. These results indicate that no significant interactions occurred during the preparation of solid dispersion [32].

#### 3.1.5. Transmission Electron Microscopy Imaging

**Figure 5:**
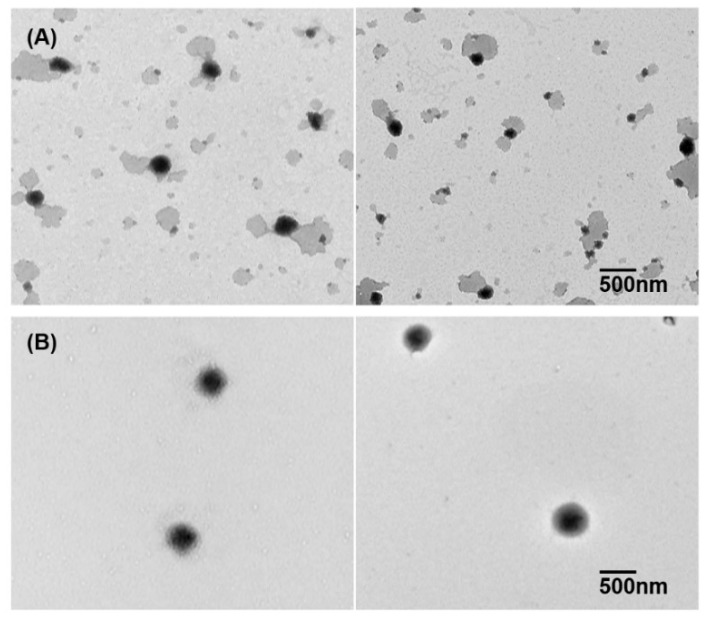
Transmission electron microscope image of (**A**) Soluplus^®^ polymeric micelles without fluorometholone and (**B**) fluorometholone–loaded polymeric micelles (1:15).

TEM imaging was performed to observe the morphology of FML solid dispersion within PBS. As shown in Figure 5, the solid dispersion with FML formed very fine and round polymeric micelles. The polymeric micelle solution of FML was transparent and maintained its clear appearance over 6 months. These findings support the good physical stability of polymeric micelles.

#### 3.1.6. Measurement of Solubility of Fluorometholone

**Figure 6:**
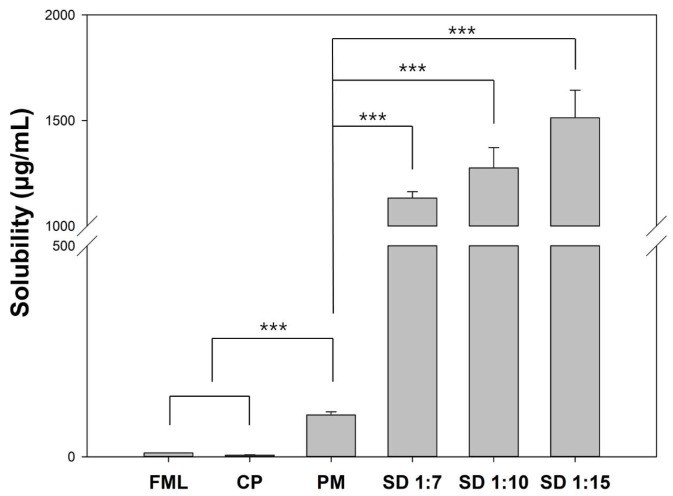
Phase solubility of fluorometholone in various formulations. Student’s *t*-test was performed to compare statistically significant differences of solubility and *** indicates *p* < 0.001.

The solubility of pure FML, physical mixture, and solid dispersions was evaluated. Pure FML showed poor intrinsic solubility (8.92 ± 0.01 μg/mL). The solubility of FML from physical mixture and solid dispersions was much higher than that of pure FML. Solid dispersions showed approximately 169.6-fold higher solubility than pure FML. In addition, the solubility of solid dispersions of 1:7, 1:10, and 1:15 formulations was approximately 11.4-, 12.9-, and 15.3-fold higher than that of physical mixture, respectively (Figure 6).

### 3.2. Ex Vivo Permeation Study

**Figure 7:**
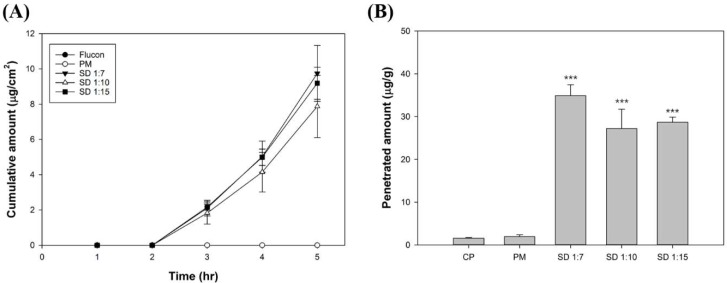
(**A**) Cumulative permeated amount of fluorometholone through porcine corneal tissue and (**B**) deposited amount of fluorometholone within porcine corneal tissue. Student’s *t*-test was performed to compare statistically significant differences of the distributed amount of drug with commercial product (CP) and physical mixture (PM). *** represents *p* < 0.001.

Porcine corneal tissues were used to evaluate the permeation and penetration of FML in various formulations. In the case of commercial product and physical mixture, FML was not detected from the receptor chamber (Figure 7A), and a relatively small amount accumulated in the tissues (Figure 7B). FML in polymeric micelles began to permeate the porcine corneal tissues after a lag time of 2 h. The *J_s_* values of FML from 1:7, 1:10 and 1:15 formulations were 3.82 ± 0.47, 3.02 ± 0.47 and 3.51 ± 0.30, respectively. There was no statistically significant difference in *J_s_* between SD groups. The accumulated amount of FML from 1:7, 1:10, and 1:15 formulations in porcine corneal tissue after 5 h was 34.89 ± 2.08, 28.89 ± 4.31, and 28.68 ± 0.98 μg/g, respectively (Table 3). The amount of FML from 1:7 formulation deposited within the porcine cornea was around 22.4-fold and 18.0-fold higher than that from commercial product and physical mixture, respectively, and the 1:7 formulation showed the highest deposition. 

#### Imaging Study Using Coumarin-6 as a Fluorescent Probe

**Figure 8:**
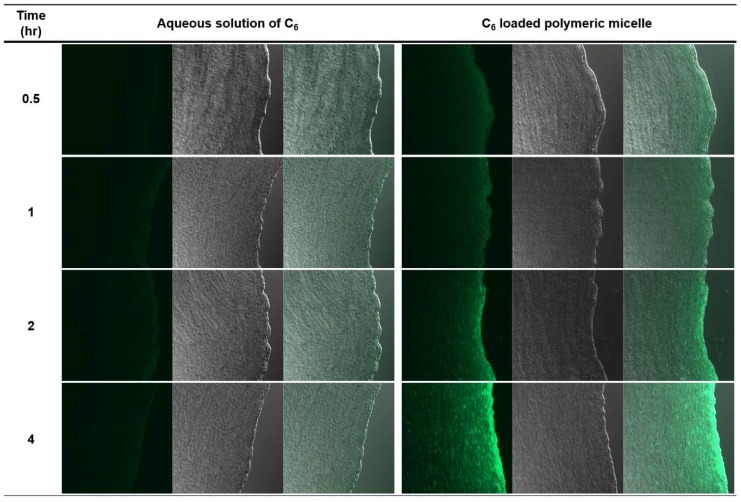
Confocal scanning laser microscopy image of porcine corneal tissues after the application of C_6_ aqueous solution and C_6_–loaded polymeric micelle solution at predetermined time points (Left: fluorescence; Center: bright field; Right: overlay). The magnification was X10.

Confocal laser scanning microscope was used to confirm the corneal permeation enhancing effect of polymeric micelles of Soluplus^®^ using C_6_ as a model compound of poorly water-soluble drugs. After treatment of C_6-_loaded polymeric micelle (C_6_PM) and aqueous solution of C_6_ (C_6_AS), C_6_ in porcine corneal tissues was observed at various time points (Figure 8). After 0.5 h, both C_6_PM- and C_6_AS-treated group showed green fluorescence on the surface and corneal epithelium. The fluorescence intensity was stronger in C_6_PM-treated group. In C_6_PM-treated group, fluorescence intensity shifted to the inner side of corneal tissue over time, and the fluorescence intensity on the epithelium was concentrated. However, in case of C_6_, fluorescence was retained only on the surface and epithelium with a weak intensity.

## 4. Discussion

The purpose of this study was to solubilize poorly water soluble FML using Soluplus^®^ to prepare eye drop formulation. Soluplus^®^ is a recently introduced new polymer that can increase the solubility of poorly water-soluble drugs. It has an amphiphilic structure that self-assembles and forms stable polymeric micelles in aqueous environment. The safety of Soluplus^®^ has been evaluated through in vitro toxicity test and in vivo ocular tolerance test, and the results showed the feasibility of use of Soluplus^®^ in ocular drug delivery systems [34]. Because of the low CMC of 7.6 mg/L, Soluplus^®^ can increase the solubility of poorly water-soluble drugs at low concentrations [19,35]. For example, Pluronic^®^ F-127, a block copolymer surfactant that is widely used for the solubilization of various poorly water-soluble drugs, is also used for ocular drug delivery. However, it has a CMC of 2.8 × 10^−6^ M [36], which is higher than that of Soluplus^®^, and therefore requires a higher concentration to solubilize poorly water-soluble drugs. Considering the high CMC of poloxamer, which is used as a solubilizer, Soluplus^®^ can be considered a good candidate for solubilization and delivery of poorly water-soluble drugs to the eye safely.

**Figure 9:**
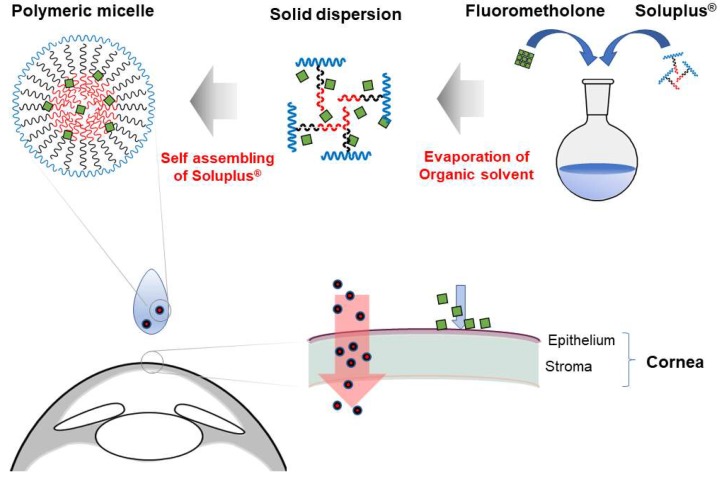
Schematic representation of the action mechanism of fluorometholone-loaded polymeric micelle. Fluorometholone-loaded solid dispersion was successfully prepared. The solid dispersion spontaneously forms polymeric micelles within the water phase. The nano-sized polymeric micelle enabled the permeation of water insoluble fluorometholone with high efficiency. In contrast, fluorometholone dispersed in commercial eye drop product has very low corneal permeation efficiency. The large, pink arrow indicates high permeation and deposition, while the small and blue arrow shows low permeation and deposition.

The schematic representation of the main concepts of this study is shown in Figure 9, and its content can be described as follows. FML-loaded polymeric micelles used in this study were prepared by simple solvent evaporation and rehydration methods. For each formulation, the concentration of FML used was 0.1% (*w*/*v*), which is equal to that of commercial suspension product. Suspension is defined as a state in which small insoluble solid drug particles are dispersed in an aqueous solution, and it generally has an opaque appearance. 

To date, various methods have been used to solubilize FML; however, no method capable of sufficiently dissolving FML has been developed. Malaekeh-Nikouei et al used cyclodextrin derivatives to solubilize FML. In their study, the solubility of FML by 5% β-CD, 5% γ-CD, 5% HP-β-CD, and 5% HP-γ-CD was approximately 0.5 mg/mL. The maximum solubility of FML (1.16 ± 0.04 mg/mL) was achieved by 5% SBE-β-CD. Considering that the measured intrinsic water solubility of FML was only 8.92 ± 0.01 μg/mL, the 1:15 polymer micelle system showed a solubility of 1.51 ± 0.11 mg/mL. Moreover, the solubility increased approximately 169.6-fold compared with that by raw materials, and it was 1.3-fold higher than that by pure SBE-β-CD [17].

**Figure 10:**
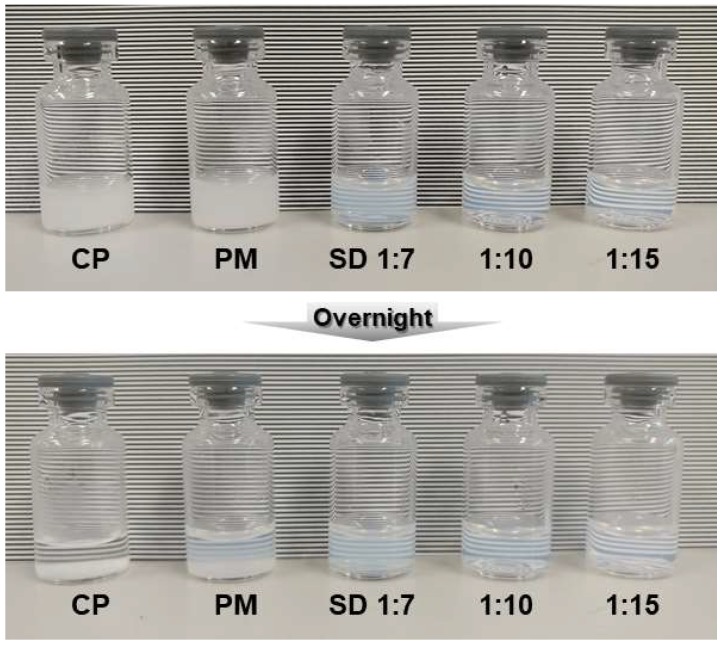
Appearance of commercial product, physical mixture (PM), and polymeric micelle formulation prepared by solid dispersion process. The commercial product sedimented when left overnight and PM showed slight sedimentation. However, all the polymeric micelle formulations employing solid dispersion showed transparency and good physical stability.

The polymeric micelle systems in the present study were transparent because of complete dissolution of FML. As can be seen from Figure 10, the commercial product was sedimented within 12 h and had to be shaken before use and used immediately. However, the polymeric micelle systems in this study showed a very clear appearance with enhanced physical stability. Transparency can be used as a factor to distinguish the polymeric micelle system from the commercial product, and patients can be dosed correctly without mixing of samples.

Particle size measurement showed that the commercial product had a much larger particle size and uneven distribution than the polymeric micelles. This indicates that FML within the commercial product is not homogeneously dispersed or dissolved in aqueous solution. Therefore, the commercial product has difficulty in controlling the dose accurately. In addition, large particles can cause adverse effects such as eye irritation, blurred vision, and redness. In contrast, polymeric micelles showed uniform size distribution with small particle size and were transparent because of sufficient dissolution of FML. Thus, FML-loaded polymeric micelle can be a potential strategy to improve the adverse effects of FML suspension.

The physicochemical characteristics were determined by DSC, PXRD, and FT-IR spectroscopy. The results showed that FML was in crystalline form in commercial product and physical mixture. However, the crystalline form of FML was converted to amorphous form in solid dispersion, with no significant chemical interactions between FML and Soluplus^®^. The solubility of the drug was enhanced because the amorphous form has lower lattice energy than the crystalline form [32,37].

The solubility enhancing effect of solid dispersions was evaluated by solubility test. As the amount of Soluplus^®^ increased, the amount of drug encapsulated in the micelle or matrix increased. In the physical mixture, Soluplus^®^ increased the solubility of FML slightly. In contrast, Soluplus^®^ increased solubility not only by offering matrix for solid dispersion, but also by forming polymeric micelle spontaneously [23]. The synergistic effect of these two mechanisms showed high solubility enhancement of FML in aqueous media.

The corneal permeation of FML was evaluated by ex vivo permeation study. FML in commercial product and physical mixture did not pass through the porcine corneal tissue for 5 h, and a small amount was accumulated in corneal tissue. However, in the solid dispersions, FML permeated porcine corneal tissues after a lag time of 2 h. The accumulated amount of FML by 1:7, 1:10, and 1:15 formulations in the corneal tissue was 22.4-, 18.5-, and 18.4-fold higher than that by the commercial product, respectively. Less than 1% of FML was accumulated in the corneal tissue from the commercial product and physical mixture. However, in the case of solid dispersions, approximately 3% of loaded FML permeated the corneal tissue and around 10% was accumulated in the corneal tissue. The permeation enhancing effect of polymeric micelle was also observed in C_6_ permeation study. C_6_ incorporated in polymeric micelle showed higher permeation in the porcine corneal tissue than aqueous solution. This is because as the drug dissolves at higher concentrations, polymeric micelle can increase the permeability of the drug by creating a higher concentration gradient for the barrier [23]. Also the temperature dependent rheological property of Soluplus^®^ is possible mechanism for permeation enhancement. Soluplus^®^ change from liquid to weak gel phase temperature dependently on the ocular surface. Thus, the residence time of drug can be increased, which has a beneficial effect on enhanced corneal permeation of FML [26].

## 5. Conclusions

This is the first report on the use of Soluplus^®^ for the preparation of an eye drop formulation of FML with enhanced water solubility, transparency, and physical stability. Through various characterization studies, it was confirmed that FML was converted from crystalline form to amorphous form in solid dispersion, and its solubility was increased by this mechanism. In addition, polymeric micelle formation resulted in small and uniform particles, and showed high porcine corneal tissue permeation property. Thus, polymeric micelle formulation method employing solid dispersion technique can be a promising strategy to overcome side effects and problems, such as eye irritation and difficulty in precise dosage control in conventional suspension formulations. It is also expected to improve the compliance of patients by lowering the dose and frequency of the drug by improving bioavailability. In conclusion, solubilization with Soluplus^®^ can be a good alternative to conventional eye drops.

## Figures and Tables

**Table 1:** General ocular drug delivery systems available for pharmaceutical application.

Classification	Types	General Description
Conventional systems	Topical liquid/solution eye drop	Immediately active Concentration rapidly decline after administration, following first order kinetics Various additives added: viscosity enhancers, permeation enhancers & cyclodextrins
	Emulsion	Improvement in solubility & bioavailability Oil in water (o/w), water in oil (w/o) systems commercially exploited as vehicles for active pharmaceuticals o/w is common & widely preferred over w/o system. o/w has less irritation and better ocular tolerance
	Suspension	Dispersion of finely divided insoluble API in an aqueous solvent Suspension particles retain in precorneal pocket and improve drug contact time and duration of action relative to drug solution Dysgeusia, ocular irritation, nasopharyngitis adverse events were observed
	Ointment	Mixture of semisolid & solid hydrocarbon (paraffin) that has melting point at physiological ocular temperature (34 °C) Ointments help to improve ocular bioavailability and sustain the drug release
Nano-Technology based systems	Nanomicelle	Most commonly used carrier systems to form API in to clear aqueous solutions Amphiphilic molecules like surfactants or polymer are used to form nanomicelle High drug encapsulation capability, ease of preparation, small size, hydrophilic nanomicellar corona forming aqueous solution Micellar formulation enhance bioavailability of API in ocular tissues
	Nanoparticle	Colloidal carrieres containing size range of 10–1000 nm Nanoparticles can form nanocapsules or nanospheres Small size, leading to low irritation and sustained release property avoiding frequent administration
	Nanosuspension	Colloidal dispersion of submicron drug particles stabilized by polymers or surfactants Sterilization advantages, ease of eye drop formulation, less irritation, precorneal residence time increase and enhancement in ocular bioavailability for drugs insoluble in tear fluid.

**Table 2:** Particle size, poly dispersity index and encapsulation efficiency of various FML formulations.

Formulation	Particle Size (nm)	PDI	Encapsulation Efficiency (%)
Soluplus	64.6 ± 1.3	0.016 ± 0.011	NA
SD 1:7	102.3 ± 0.9	0.094 ± 0.029	99.63 ± 0.03
SD 1:10	95.1 ± 0.6	0.096 ± 0.020	99.67 ± 0.02
SD 1:15	74.9 ± 0.7	0.040 ± 0.004	99.72 ± 0.04
PM	225.3 ± 5.7	0.385 ± 0.006	1.48 ± 0.02
CP	2462.8 ± 190.0	0.220 ± 0.056	NA

Abbreviation: NA, not available.

**Table 3:** Permeated and deposited amount of fluorometholone in *ex vivo* porcine corneal permeation study.

Formulation	Permeated Amount (μg/cm^2^)	Steady State Flux (μg/cm^2^·s)	Deposited Amount Per Gram of Porcine Corneal Tissue (μg/g)
CP	ND	ND	1.56 ± 0.15
PM	ND	ND	1.94 ± 0.35
SD 1:7	9.74 ± 1.29	3.82 ± 0.47	34.89 ± 2.08
SD 1:10	8.98 ± 2.29	3.02 ± 0.47	28.89 ± 4.31
SD 1:15	9.19 ± 0.74	3.51 ± 0.30	28.68 ± 0.98

**Abbreviations:** CP, commercial product; PM, physical mixture; SD, solid dispersion; ND, not determined.
